# Quantifying the impact of wildfire smoke on solar photovoltaic generation in Australia

**DOI:** 10.1016/j.isci.2023.108611

**Published:** 2023-11-30

**Authors:** Ethan Ford, Ian Marius Peters, Bram Hoex

**Affiliations:** 1 School of Photovoltaic and Renewable Energy Engineering, UNSW Sydney, Sydney, NSW 2052, Australia; 2 Forschungszentrum Jülich GmbH, 91058 Erlangen, Germany

**Keywords:** Applied physics, Engineering

## Abstract

The 2019–20 Australian wildfires caused extreme haze events across New South Wales (NSW), which reduced photovoltaic (PV) power output. We analyze 30-min energy data from 160 geographically separated residential PV systems in NSW with a total capacity of 312 kW from 6 Nov 2019–15 Jan 2020. The observed mean power reduction rate for PV energy generation as a function of the fine particulate matter (PM_2.5_) concentration is 13 ± 2% per 100 μg/m^3^ of PM_2.5_. The resulting energy loss for residential and utility PV systems is estimated at 175 ± 35 GWh, equating to a worst-case financial loss of 19 ± 4 million USD. We found the relative impact to be most significant in the mornings and evenings, which may necessitate the installation of additional energy storage. As PV systems are sensitive to smoke and become ubiquitous, we propose employing them to support wildfire detection and monitoring.

## Highlights

• Smoke from Australia’s 2019-2020 wildfires reduced solar PV energy generation
• PV system revenue in NSW decreased by 19 ± 4 million USD during the wildfire period
• Mean smoke-induced PV losses are small but energy storage may benefit acute impacts
• Residential PV systems could serve as a wildfire detection and monitoring network

## Graphical abstract

**Figure:**
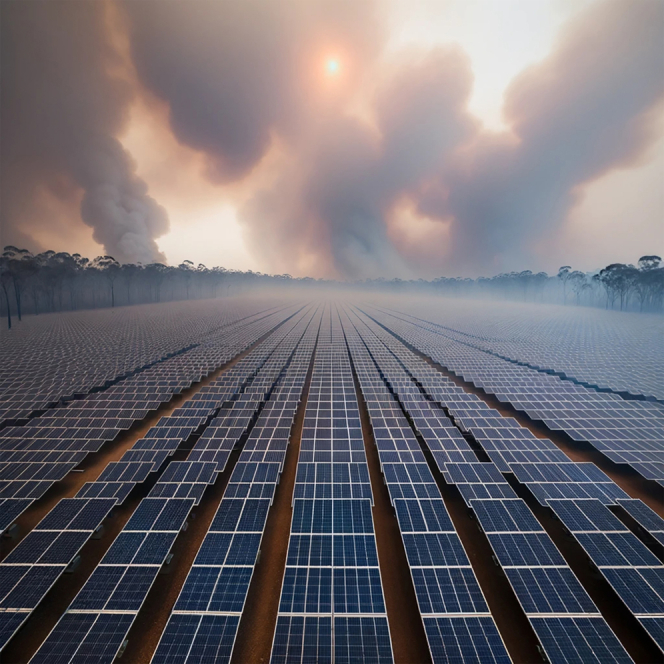

## Introduction

**Figure 1:**
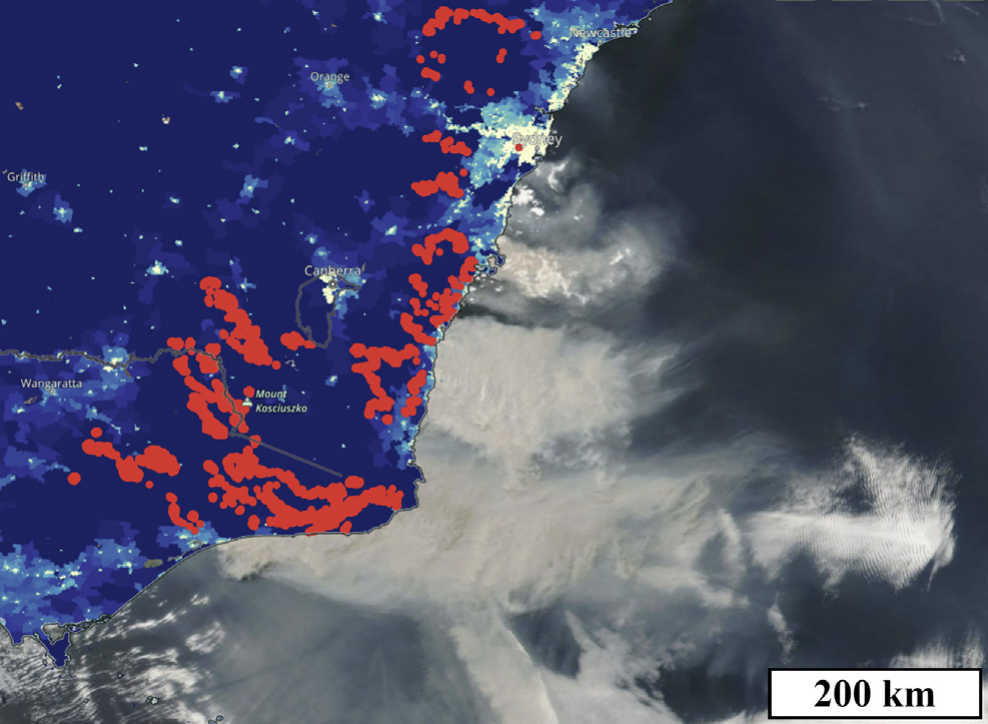
Extent of the wildfire smoke on a clear sky day NASA true-color corrected reflectance satellite image of New South Wales (NSW) showing wildfires (red) and smoke off the southeast coast of the state on 4 Jan 2020.^22^ The yellow-blue color scale on the land indicates the population density. The shown smoke event occurred on a day with minimal clouds—some are visible in the bottom-right of the image—and is an ideal example of the impact of smoke on photovoltaic (PV) performance that we wish to explore in this study. The unidirectional flow of smoke downwind, which in this case is easterly to southeasterly, highlights the importance of the location of PV systems relative to the fires.

**Figure 2:**
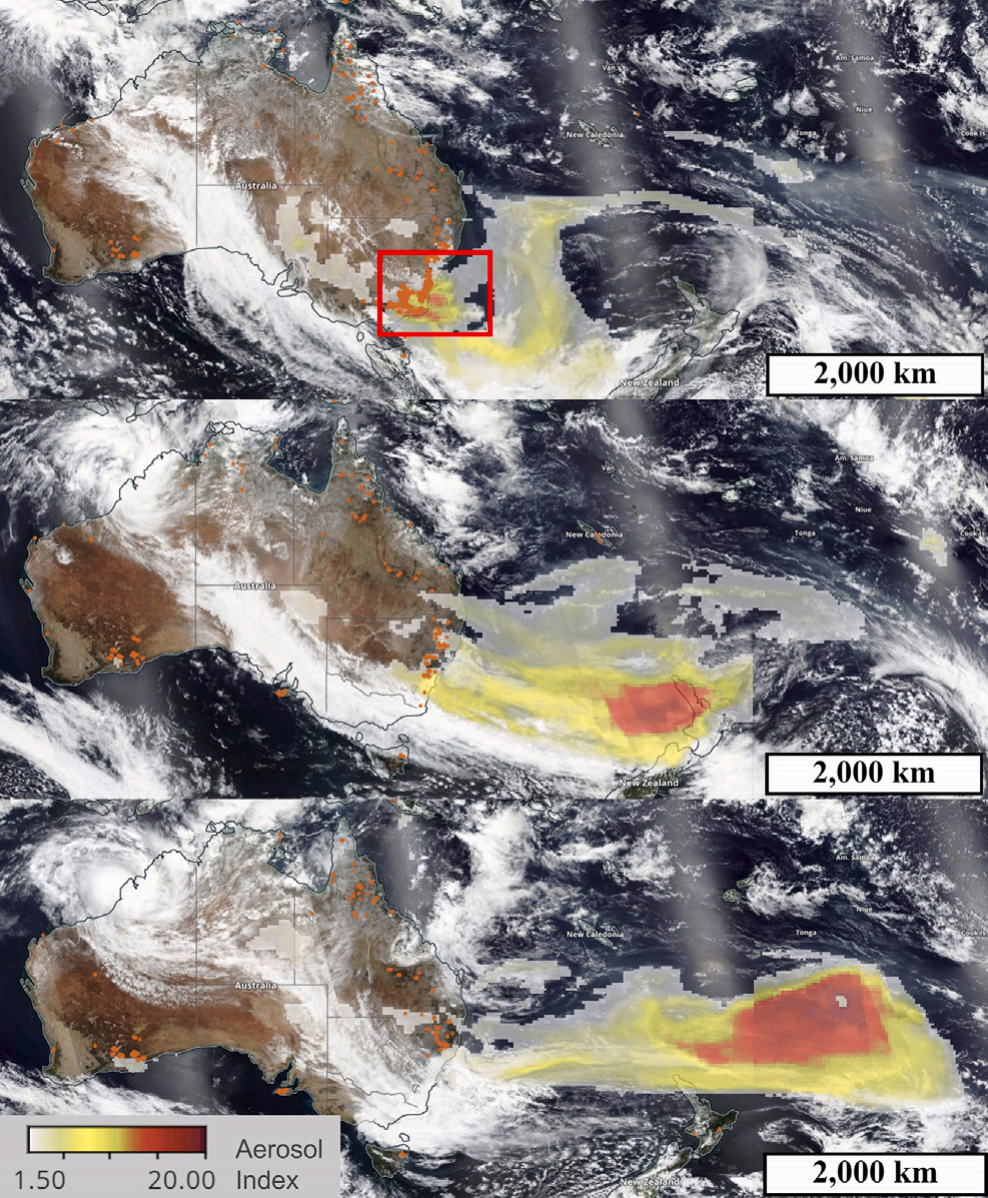
Evolution of pyrocumulonimbus clouds caused by the wildfires Satellite images of the Aerosol Index (pyrocumulonimbus layer) which indicates the presence of ultraviolet (UV)-absorbing aerosols in the atmosphere over the southwest Pacific Ocean on 4 Jan (top), 5 Jan (middle) and 6 Jan (bottom) 2020.^22^ The red square in the top image outlines the area of Figure 1. The Aerosol Index is a unitless measure related to aerosol optical depth (AOD).^28^ Here it observes dense pyrocumulonimbus events high in the troposphere and into the stratosphere, which are formed from smoke from intense wildfires. The increase in the area of the colored sections, and the prominence of red in the bottom image indicate it increased over the three days as the smoke transformed into immense clouds. At over 4,000 km in length on 6 Jan 2020, the cloud event is comparable in size to the width of Australia. Orange dots indicate the daytime fires and thermal anomalies for each day, showing the intense fires on 4 Jan 2020 in the southeastern tip of Australia which led to the enormous smoke plumes that traveled across the oceans over the following days. This helps us to visualize the mass and volume of the Australian wildfire smoke which traveled to South America.

The 2019–20 Australian wildfires were a natural disaster of national and even global proportions. The fire burnt an estimated area of 243,000 km^2^, led to the loss of 33 lives, killed countless animals, destroyed thousands of buildings, and released more than 700 million tonnes of CO_2_ into the atmosphere.^1^^,^^2^^,^^3^^,^^4^ Areas not directly affected by fire were often subject to extreme smoke, haze, and poor air quality, with strong winds carrying heavy smoke thousands of kilometers (Figures 1 and 2). In January 2020, smoke from the Australian wildfires traversed the Pacific Ocean and reached the stratosphere over Punta Arenas, Chile.^5^ Wildfire smoke consists predominantly of fine aerosol particulate matter less than 2.5 μm in diameter (PM_2.5_).^6^ It poses serious negative health implications, with ambient air pollution responsible for 4.2 million premature deaths globally in 2016, according to the World Health Organization.^7^ Borchers Arriagada et al. estimate that smoke from the Australian wildfires contributed to 417 excess deaths and 4,456 hospitalizations in eastern Australia.^8^

Recent studies have shown the empirical relationship of fire regimes is breaking due to climate change.^9^ The 2019-20 Australian wildfires were scale-invariant with many outbreaks of small and large fires.^9^ This led to an abrupt and pronounced increase in fire scale, intensity, and the average area burned.^10^ Frequent fires of this type could limit regrowth and transform the vegetation environment, such as from temperate forest to forest-savannah, as shown for areas of Canada and Australia which are closely approaching a phase transition.^9^^,^^10^^,^^11^ Consequently, increases in fuel dryness, fuel load, and thus smoke production from wildfires are likely in the short to medium term.^12^

In addition to these catastrophic effects on lives and environment, wildfires have various secondary effects, including the reduction of solar energy generation. Wildfire smoke attenuates solar irradiance and leads to soiling via the deposition of particles on the solar modules’ surfaces. The reduction in irradiance decreases the electric energy yield of PV systems and is thus of potential concern with respect to reliability and commercial sustainability of PV installations. PV power plays a central role in Australia’s move toward carbon emissions reduction. The PV capacity in Australia was 16.2 GW in Jan 2020, has grown to surpass 28.2 GW as of Sep 2022, and is projected to reach 80 GW by 2030.^13^^,^^14^ Australia has the highest per capita PV capacity of any nation with 1.049 kW/capita in 2021, over half of which is rooftop PV.^15^ Extreme wildfires, on the other hand, are a growing issue due to climate change.^16^^,^^17^ Hence, a thorough and accurate understanding of how exactly wildfires impair PV output is vitally important for ensuring the service security of the future Australian power grid. Moreover, wildfires are not only a growing concern for Australia, but in many parts of the world, as recent and dire examples in France, Spain, and California show.^18^^,^^19^^,^^20^^,^^21^ A study of the impact of wildfires on PV power is, hence, of global significance.

The impact of particulate matter in the atmosphere on photovoltaic performance has been observed on a number of occasions. Anthropogenic air pollution, most likely caused by cars and coal fires in Delhi, was observed to reduce sunlight intensity by 12.5% ± 1.4% for every 100 μg/m^3^ of PM_2.5_ particle concentration. This observation was based on data from a single PV installation and a nearby air quality sensor.^23^

Poor air quality and haze originating from wildfires in Sumatra resulted in a 15–25% reduction in PV system yield in neighboring Singapore based on observations from ten research installations.^24^ A fire burn event on a clear sky afternoon in Canberra in 2014 resulted in peak reductions in PV generation of 27% at the test site.^25^ More recently, focus has shifted to understanding wildfires in California and their impact on solar energy production. One study found a reduction in normalized PV generation of 9.4–37.8% when PM_2.5_ ranged from 50 to 200 μg/m^3^ based on combined regression modeling and observations from 68 PV installations.^26^ A second study found a reduction in the PV yield of 9–49% for an aerosol optical depth (AOD) of 0.5–4.5.^27^ AOD is the measure of absorbing and scattering aerosols in the atmosphere calculated from the wavelength-dependent extinction of light typically at a wavelength of 550 nm.^28^ An AOD of zero represents a clear day with no aerosols. Additionally, smoke created by wildfires in the Western United States (US) caused a reduction in the PV yield that varied with geographical position. The worst affected locations saw a mean reduction in the PV yield of up to 15% for the hour of 12 p.m.–1 p.m. across the Californian fire season based on a model using satellite data.^27^ Interested readers are referred to a recent review by Sadat et al. that summarizes the impact of haze on PV performance.^29^

In this work, we present our analysis of the impact of wildfire smoke on PV installations, based on measurements for 160 residential PV installations and 17 particulate matter sensors distributed across NSW. The presented analysis builds upon the self-referencing method described by Peters et al.^23^^,^^30^ We improved the previous analysis by including a significantly larger number of sites, enabling a statistical analysis with respect to power reduction, time of day, and distance between site location and PM_2.5_ measurement station that was not possible in the previous studies. Furthermore, we add an air mass (AM) correction approach, which is necessary to correctly interpret results under oblique incidence. In contrast to Gilletly et al. and Donaldson et al. our method relies exclusively on data from ground measurements, and we assess the relationship of distance between sites on correlation strength.^26^^,^^27^ The analysis by Gilletly et al. relies on a regression model using multiple environmental parameters.^26^ With a modelling-based approach, it is challenging to distinguish between dimming and soiling, as Gilletly et al. also report. This issue is greatly reduced with self-referencing, as soiling also affects the reference. In addition, we were able to access PM_2.5_ measurement stations in closer proximity (less than 5 km) to the PV installations than used by Gilletly et al., resulting in greater correlation between measured PM_2.5_ concentration and PV output reduction.^26^ The study by Donaldson et al. correlates PV power output to AOD.^27^ This approach is effective at explaining the overall impact of atmospheric conditions, but is limited with respect to single events like wildfires, as AOD and PM_2.5_ have been found not always to correlate.^23^ Beyond the presented technical analysis, we extend our analysis to the broader impact of wildfires on the economic and societal benefits of PV power generation in Australia and the world.

The below sections detail the datasets, analysis processes and statistical results regarding the effects of wildfire-induced PM_2.5_ on residential PV system production. First, we show how we selected the 160 residential rooftop PV sites out of the available 710 for studying the impact of PM_2.5_ concentration on PV performance. Subsequently, we discuss how we calculate the relative PV performance as a function of the PM_2.5_ concentration by self-referencing periods without significant PM_2.5_ concentration. Then we will present the aggregated results for the 160 sites and calculate the PV performance attenuation factor. Finally, we use this information to calculate the economic impact of the Australian Black Summer Wildfire season and discuss the wider impact of this work.

## Results

### Air mass correction

The attenuation of sunlight by wildfire smoke is expected to follow Beer-Lambert’s law. The path length of the sunlight through the atmosphere will vary with the solar zenith angle, which depends on the time of day and year. This path length is analogous to AM—the volume of air along a given line of sight, such as between the PV system and the sun. The PM_2.5_ concentration represents the number of PM_2.5_ particles per unit volume of air at the altitude of the PV system. By extrapolating this per-unit concentration along the line of sight, the sunlight will encounter more PM_2.5_ particles if it travels through more atmosphere. Thus, for a given concentration of smoke particles, the reduction in sunlight intensity will be greater for increased AM (i.e., increased path length during oblique incidence). We corrected for AM by multiplying each PM_2.5_ data point with the AM at the time, location and altitude of measurement:

(Equation 1) $${PM}_{2.5,AM}=AM\cdot {PM}_{2.5}$$

where ${PM}_{2.5,AM}$ is the AM-corrected PM_2.5_ concentration in μg/m^3^, AM (unitless) is the air mass recorded at the midpoint of the period of the PM_2.5_ measurement, and ${PM}_{2.5}$ is the measured PM_2.5_ value in μg/m^3^.

### Relative power reduction rates

**Figure 3:**
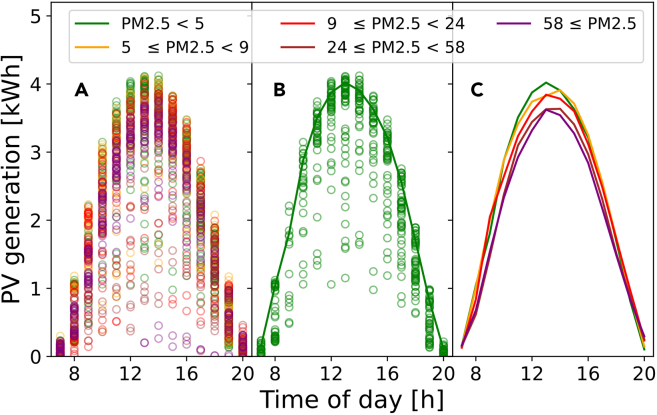
Extracting cloud-free PV generation for various PM_2.5_ ranges using the 80-percentile filter Creation of clear sky curves. (A) Raw binned data; (B) 80-percentile; (C) repeating step ‘(B)’ for all PM_2.5_ ranges (μg/m^3^).

**Figure 4:**
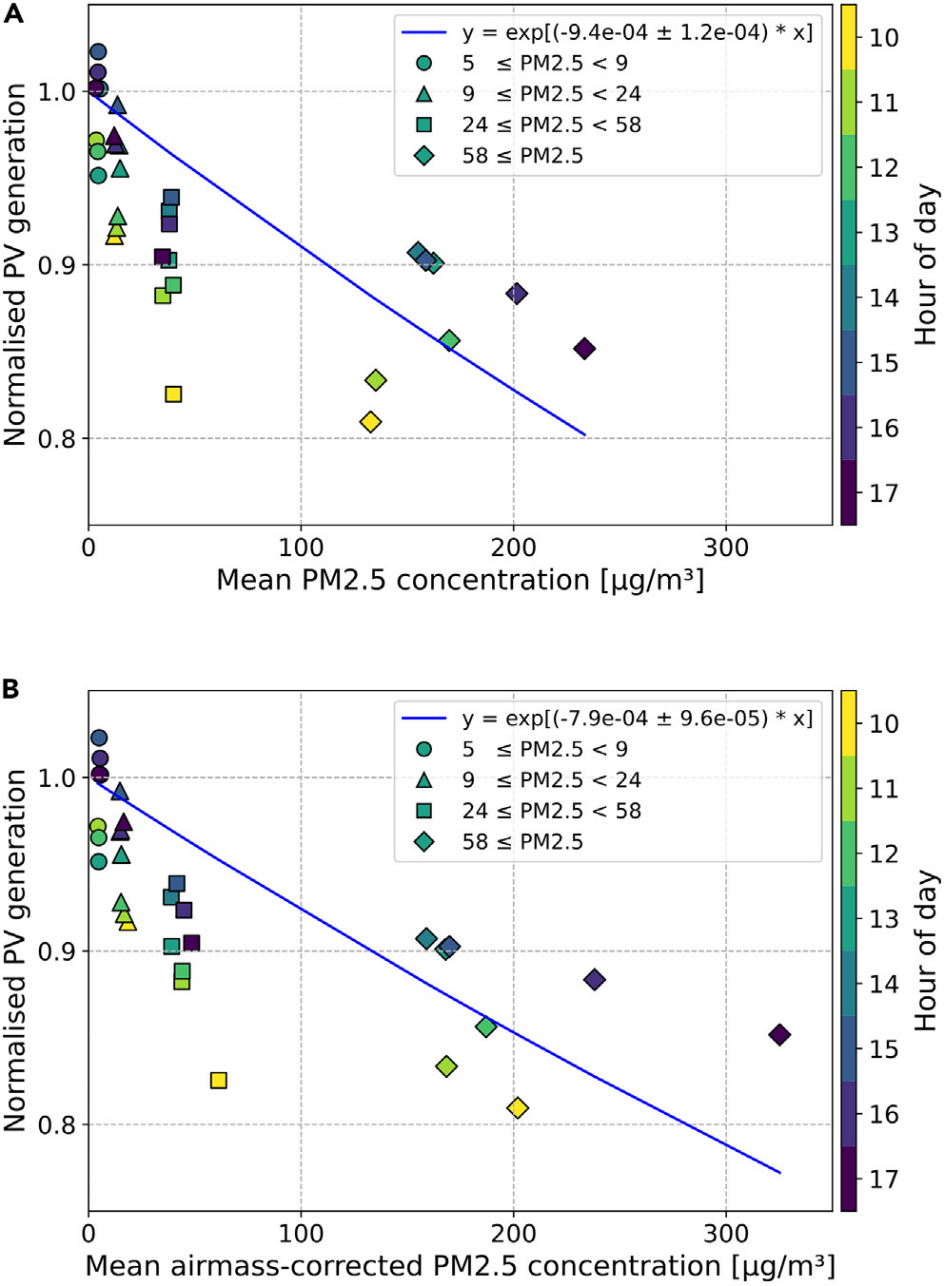
How air pollution reduces PV generation for a single PV site over the course of a day (A) Normalized PV generation by hour (from 80-percentile filter) and PM_2.5_ range (μg/m^3^) plotted against the mean PM_2.5_ concentration for those measurements. (B) Normalized PV generation plotted against the air mass-corrected PM_2.5_ concentration—equivalent to the product of air mass and PM_2.5_.

PV generation was normalized to negligible air pollution (the green curve of Figure 3) for each hour and the results for a single PV site are shown in Figure 4A showing the trend in normalized generation for each hour of the day (color bar). It can be seen that the normalized PV generation in the morning was significantly lower than for later times with the same PM_2.5_ concentration. We subsequently corrected for atmospheric optical path length (air mass), and the results are shown in Figure 4B. We can see this AM-correction pulls the data points along the x axis to the right, which significantly reduces the spread of the data along the exponential fit. The reduction rate is less severe than the uncorrected data in Figure 4A because this method removes greater PV power reductions due to the sunlight traveling through more smoke via more atmosphere instead of a higher PM_2.5_ concentration. For this PV site, we see that the early morning results show the largest reduction in normalized PV generation as a function of the AM-corrected PM_2.5_ concentration. Across the 160 PV systems analyzed, the strongest reductions were seen in the mornings and evenings, with the least impact around 1p.m. (see Figure S1 which depicts an hour-specific relative reduction rate). A complete explanation for this symmetrical impact is not yet understood—the AM-correction may be incomplete, or daily wind and weather patterns may play a role. Beer-Lambert’s Law was fit to the data to find the mean relative reduction rate:

(Equation 2) $$\frac{P\left({PM}_{2.5}\right)}{P\left(0\right)}=\exp\left(-R\cdot {PM}_{2.5,AM}\right)$$

where $P\left({PM}_{2.5}\right)$ is the measured PV energy production in Wh, P(0) is the energy production for zero air pollution in Wh, and R is the relative reduction rate for the PV system in m^3^/μg.

**Figure 5:**
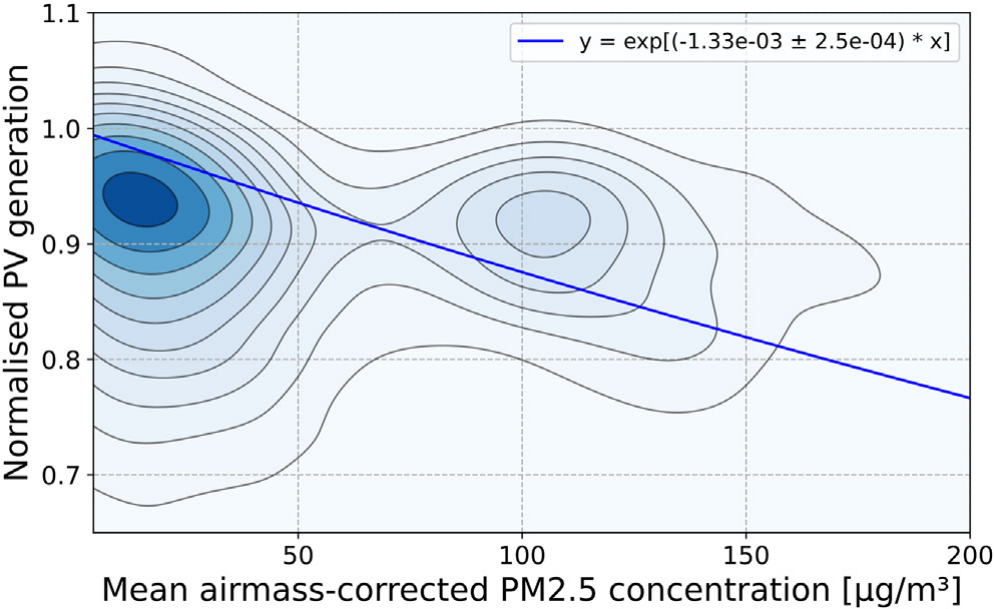
How air pollution reduces PV generation for 160 PV sites in NSW 2D kernel density plot of normalized PV generation for all PV sites as a function of the AM-correct PM_2.5_ concentration including the fit of Beer-Lambert’s Law (blue line) and the corresponding equation in the top-right of the chart. The darker blue area indicates a higher density, or greater number of data points. The contour lines are spaced logarithmically to help show the distribution in areas of lower density.

The above process was repeated for all PV sites and the results are plotted in Figure 5 in the form of a blue gradient 2D kernel density. The darker blue gradient on the plot indicates the highest density of data points are located around an AM-corrected PM_2.5_ concentration of less than 20 μg/m^3^ and a normalized PV performance of 0.94. There is a small but noticeable increase in density located between 85 and 120 μg/m^3^ and 0.87–0.95. Smoky days were sporadic and the baseline air quality of NSW was generally very good with most PM_2.5_ measurements below 30 μg/m^3^. Yet, large spikes in concentration to well above 500 μg/m^3^ occurred for particular sites during extreme haze events which likely contributes to the secondary peak of this bimodal distribution. As a result, Beer-Lambert’s law (blue line of best fit in Figure 5) underestimates the impact for low PM_2.5_ concentrations, but overestimates for high concentrations. The value for R was found to be (1.33 ± 0.25) · 10^−3^ m^3^/μg in this study, which is almost identical to the 12.5% reduction in light intensity per 100 μg/m^3^ determined in the previous work of.^23^

### Correlation between distance and reduction rate

**Figure 6:**
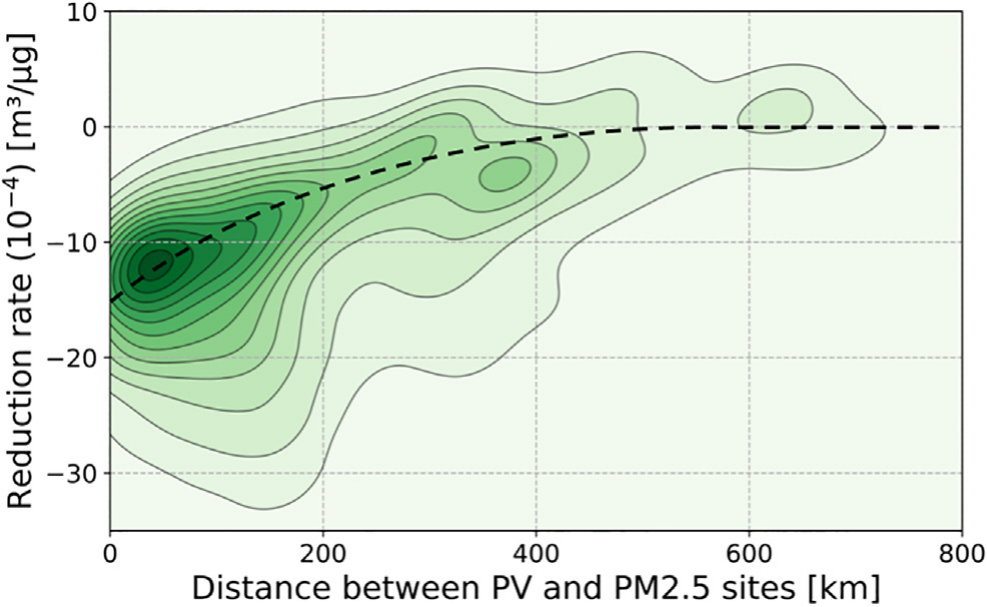
Effect of distance on the correlation between PM_2.5_ and PV generation This 2D kernel density plot illustrating the impact of air pollution on PV generation was calculated for all possible combinations of 537 PV systems and 30 PM_2.5_ sites across NSW with at least 95% data completeness. Larger negative reduction rates indicate that PM_2.5_ caused significant reductions in PV generation for that PV site. Reduction rates near zero indicate no correlation (noise). The correlation is centered around 12 · 10^−4^ m^3^/μg at a distance of approximately 0–100 km (darkest green area), but decays quickly and disappears beyond roughly 500 km. This supports the criteria of only selecting PV sites within a 5 km bound of PM_2.5_ monitors as these smaller distances are where the decay in correlation is the strongest. N.B. The spatial correlation in this figure is likely specific to weather conditions in NSW during the wildfires. The black dotted line is not a mathematical fit but is used as a guide for the eye.

To analyze the effect of distance between PV sites and PM_2.5_ monitors, we correlated every PM_2.5_ site with every PV site and plotted the results in Figure 6. We observed that sites that are further away show a smaller correlation between PV power and PM_2.5_ concentration than collocated sites. This is to be expected as weather patterns diffuse the wildfire smoke both spatially and temporally, as shown in Figure 2. We also found that distance alone is not a sufficient criterion for correlation. Looking at Figure 1, it is apparent that smoke can travel on a distinct path with limited dispersion. Orthogonally to the wind direction, correlations will decline quickly, and if the PV site and PM_2.5_ measurement are on opposite sides of a fire (i.e., one site downwind and the other upwind), the correlation will disappear entirely. For this reason, we limited our analysis to sites that exhibited a significant correlation—which was ensured by only selecting sites within a 5 km radius.

### Energy and financial impact

**Figure 7:**
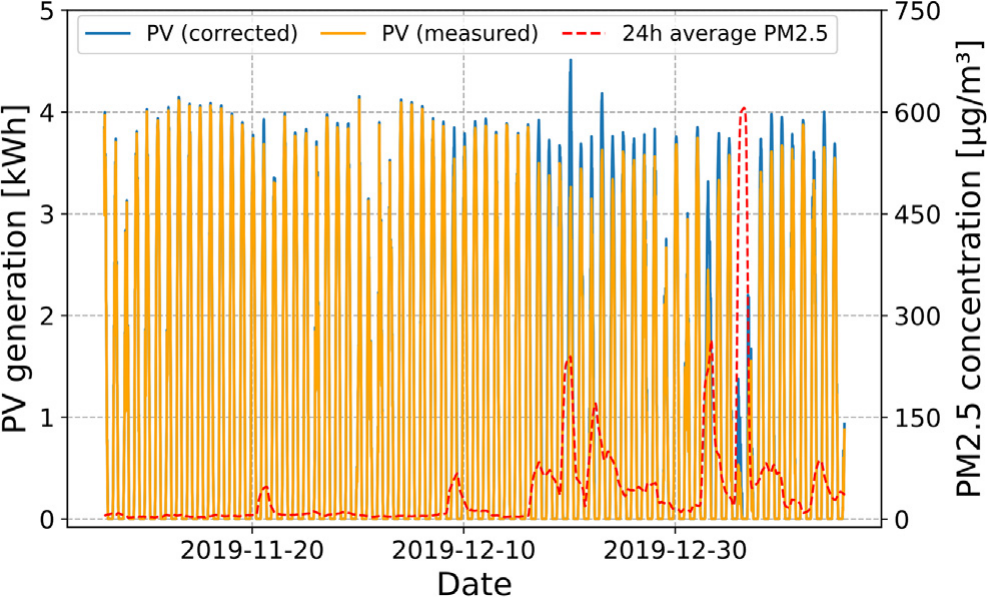
Correcting PV generation for clear sky conditions/What would PV generation have been without the wildfires Raw PV generation (orange), 24-h average of PM_2.5_ concentration (red) and PV generation as corrected for zero air pollution (blue) for the wildfire period for a PV site at Wagga Wagga.

The relative reduction rate from Equation 2 was used to correct PV generation for air pollution (i.e., calculate the performance for PM_2.5_ of 0 μg/m^3^) and thus estimate the effective PV energy loss (ΔP) due to wildfire smoke using Equation 3. This involved finding the relative difference between the sum of the measured and corrected PV energy generation time series data in Wh of each PV system (Figure 7). A 24-h moving average of ${PM}_{2.5,24h}$ was used in the back-calculation to smoothen the result, as there were major peaks in PM_2.5_ concentration with values in excess of 1,000 μg/m^3^.

(Equation 3) $$\Delta P=\frac{\Sigma P\left(0\right)\;-\;\Sigma P\left({PM}_{2.5,24h}\right)}{\Sigma P\left(0\right)}$$

Some limitations of the correction procedure are observed in mid to late Dec 2019 where the corrected PV output is significantly above that of clear sky and smoke-free days earlier in Dec. The reduction in normal PV output around the PM_2.5_ peak on 5 Jan 2020 occurred due to a large cloud band moving over NSW––see Figure 2. This coincided with intense and widespread fire on 4 Jan 2020, resulting in the very low PV output and peak PM_2.5_ concentrations around 4–5 Jan 2020.

The total installed PV capacity of NSW $\left(C_{NSW}\right)$ in Dec 2019 was approximately 5 GW: with 3 GW of residential $\left(C_{NSW,res}\right)$ and small commercial, and 2 GW of utility scale and large commercial $\left(C_{NSW,util}\right)$.^33^ The statewide PV energy loss over the wildfire period of ${\Delta P}_{NSW}$ was estimated in units of GWh using Equation 4 where C is the total PV capacity analyzed in this study of 312 kW.

(Equation 4) $${\Delta P}_{NSW}=\frac{\Delta P\cdot C_{NSW}}{C}$$

We then used Equation 5 to calculate the state-wide energy loss in GWh on a single smoky day of ${\Delta P}_{NSW,smoky}$ by dividing the state-wide energy loss by the number of days of the study (N) and multiplying by the ratio of the relative energy loss on the smoky day $\left({\Delta P}_{smoky,\%}\right)$ compared to the entire wildfire period $\left({\Delta P}_{NSW,\%}\right)$.

(Equation 5) $${\Delta P}_{NSW,smoky}=\frac{{\Delta P}_{NSW}\cdot {\Delta P}_{smoky,\%}}{N\cdot {\Delta P}_{NSW,\%}}$$

The worst-case financial loss $\left(F_{loss}\right)$ in USD assumed self-consumption of all PV generation and was calculated as per Equation 6 with a residential electricity rate $\left(R_{res}\right)$ of 0.2329 AUD/kWh.^34^ This is equivalent to 0.1609 USD/kWh using the exchange rate of 1.4476 AUD/USD on 15 Jan 2020.^35^ A utility PV plant electricity price $\left(R_{util}\right)$ of 0.03560 USD/kWh was estimated as the mean wholesale market price for Dec 2019 for the hours of 8 a.m.–6 p.m., inclusive, sourced from the Australian Energy Market Operator.^36^

(Equation 6) $$F_{loss}={\Delta P}_{NSW}\left(\frac{R_{res}{\cdot C}_{NSW,res}+R_{util}\cdot C_{NSW,util}}{C_{NSW,res}+{\;C}_{NSW,util}}\right)$$

A summary of these findings are presented in Table 1. The unweighted mean energy loss of all PV systems was 4.2 ± 0.8% for the entire period of analysis. To give a sense for the variations in this reduction, the mean energy loss for the PV system in Wagga Wagga was roughly four times that at almost 17% for a single cloudless but smoky day on 23 Dec 2019 with an average daytime PM_2.5_ concentration of 111 μg/m^3^. The maximum relative energy loss for this PV system was 42% from 7:30 a.m.–8 a.m. local time which is consistent with other real-world data collection indicating attenuation in PV performance of up to 40% according to Sadat et al.^29^

**Table 1:** Estimated energy and financial loss for all PV systems in NSW with standard deviations as error bounds

State-wide loss scenario	Energy loss [%]			Energy loss [GWh]			Financial loss [million USD]		
	Lower	Mean	Upper	Lower	Mean	Upper	Lower	Mean	Upper
71-day wildfire season	3.42	4.22	5.03	141	175	211	15.6	19.4	23.3
Smoky day [111 μg/m^3^]	13.9	16.8	19.7	8.05	9.83	11.6	0.892	1.09	1.28

By extrapolating to the total PV capacity of NSW in Equation 4 we estimate the state-wide energy impact at over 175 GWh for the wildfire period. Roughly 5.6% of this energy reduction—a loss of approximately 10 GWh—occurred on 23 Dec 2019 and showcases how wildfire smoke has a sporadic but potentially severe impact on PV energy production. These cumulative energy losses caused a 19 million USD cut to revenue over the 71 days; equivalent to 4 USD/kW of installed PV on average. Households with larger PV systems, higher electricity rates and more generous feed-in tariffs were more heavily affected. This result greatly surpasses previous estimates for PV plant revenue losses ranging from 0.78 in Delhi to 5.9–9.3 million USD in Los Angeles.^23^

The energy losses in Table 1 are expected to be insufficient to significantly impact energy security in Australia. Overall losses were 4.2 ± 0.8% over the wildfire period and less than 1% over a full year. Furthermore, residential PV systems are typically designed to maximize daily energy production during winter. Thus, they overproduce in summer when wildfires are most frequent, often resulting in clipping as the inverter capacity is lower than the sum of the DC capacity of the solar panels. This was observed in the data with over 16% of systems clipped during the period of the wildfires as shown in data filtering section. Peak losses can, therefore, at least partially, be compensated by a reduced curtailment. Furthermore, the integrated losses due to clipping likely exceed those due to haze, suggesting that the overall economic impact should be bearable. Wildfires may nevertheless pose an additional challenge for the grid; as discussed, Figure 4 indicates that losses are most significant in the morning and evening, when PV generation is ramping up and down, respectively. Reductions during those times are sensitive as high demand meets with a limited generation.^37^ Compensating for production losses here may require additional backups like battery storage.^38^^,^^39^^,^^40^

### Solar panels can monitor local air quality

It is evident that clear sky PV performance correlates with PM_2.5_ correlation. Conversely, we postulate that PV power can be used as a rough predictor for PM_2.5_ concentration. The effect of wildfire smoke on PV energy systems is geographically widespread (evidenced in Figure 1) and is more significant depending on the time of day of the smoke impact, as explained in Figure 4.

The PM_2.5_ sensors used to source the PM_2.5_ data in this study are not distributed evenly across NSW with respect to land area as observed in the figure in geospatial analysis section. It is recommended the PM_2.5_ sensors be cleaned every quarter and recalibrated once per year.^41^ In comparison, PV systems are abundant and naturally more widespread in NSW, with many systems positioned around or near population centers as shown in the figure in geospatial analysis section. Additionally, the findings in this work strongly suggest that PV systems do not need to be cleaned or maintained beyond normal maintenance for a reliable correlation between PV and PM_2.5_ to be measured. Therefore, this may provide a path to extend the capabilities of PV systems to act as proxies for air quality monitoring (AQM) devices. A sensor network consisting of PV systems would provide significant spatial resolution to support existing PM_2.5_ measurements. Utilizing PV systems to estimate local concentrations of aerosol particulates may also be more cost-effective, assuming access to live PV performance data.

PV systems could serve as an early warning detection of wildfires by providing temporal and spatial information on the presence and approximate concentrations of wildfire smoke. As such, PV systems could complement existing ground-based AQM infrastructure and satellite-derived measurements to increase our understanding of the distribution of smoke and make predictions about wildfires. This presents the opportunity to provide the community with real-time updates on air quality and how this may affect people’s health.

Furthermore, the effect may go beyond smoke; PV systems can also help make predictions about insolation, temperature and wind, but this is outside the scope of this study.^42^ One remark is there is still work necessary to see how PV systems can provide real time data, since the analyses in this work are in retrospect.

## Discussion

Wildfires are natural disasters with devastating effects, and they are becoming ever more frequent. Recent fires in Australia, California or Europe have cost lives, harmed the environment, and destroyed land and property. Apart from these calamitous effects, wildfires also reduce the power output of solar panels through dimming and soiling. Here, we investigated the impact of the Australian Black Summer Wildfires on Photovoltaic Energy Production through dimming. For the analysis, we used historic PV system energy data for 160 residential PV installations with capacities between 0.5 and 5.4 kW, and PM_2.5_ concentration data for 17 meteorological stations across NSW. We improved a self-referencing percentile data analysis technique developed by Peters et al. by adding an air mass correction, allowing the correct interpretation of data collected under oblique incidence.^23^ We used the algorithm to describe the correlation between PV system energy production and ambient PM_2.5_ concentration for the 71-day period from 6 Nov 2019 to 15 Jan 2020. By extending the analysis to 160 sites, we are able to determine the impact of time of day and distance between site location and PM_2.5_ measurement station. In contrast to predictions based on simulation, our approach intrinsically corrects for soiling when determining power losses due to wildfires.

The reduction in PV generation due to wildfire smoke is found to be 13 ± 2% per 100 μg/m^3^ for AM1.0, which is comparable to studies in Singapore, India, and the US.^23^^,^^24^^,^^26^^,^^27^ Over the course of a moderately hazy day (PM_2.5_ of 111 μg/m³), wildfire smoke reduced PV power output by 17 ± 3% for one system in Wagga Wagga. The total energy loss over the 71-day period for all 160 systems is estimated at 4.2 ± 0.8%. This corresponds to a total state-wide energy loss of 175 ± 35 GWh during the 71-day wildfire period, giving a worst-case economic loss of approximately 19 ± 4 million USD for rooftop and utility PV system owners and investors.

Overall, we assess the threat of dimming caused by wildfires to the reliability of PV production in Australia as being manageable. As wildfires mostly occur in the summer when there is a good chance for PV production being abundant, overall losses can be compensated for. The major risk is early and late in the day, when sunlight is less abundant, and we observe especially high losses. Compensating for these losses may require the installation of additional batteries. Managing this increased generation variability is also a greater financial risk than losses caused by reduced power generation. While we are not aware of reports of larger PV stations being directly damaged by the fire, the burning of infrastructure and installations constitutes a potentially severe risk.

### Limitations of the study

It should be noted the analysis utilized point measurements of PM_2.5_ concentration near the ground as a proxy for the integral of wildfire smoke through the entire atmosphere. This use of a proxy metric causes hard-to-evaluate uncertainties, yet the observed correlations, as well as results from Peters et al. and Nobre et al. indicate that estimations of PV power reduction in this way are reasonable.^23^^,^^24^ It should be noted that we did not correct for environmental factors like variations in ambient air temperature and wind speed. For the systems analyzed here, self-referencing reduces the impact of these factors, yet it cannot be excluded that systematic differences to other systems add to the uncertainty when comparing results obtained in different climates.

Recommended future work includes using PV performance data to gain a more detailed understanding of air quality during wildfires and quantifying soiling losses in the PV dataset. Furthermore, the time dependence of the smoke impact could be further investigated by identifying the cause leading to greater relative reductions in the morning and the afternoon. The long-term damages caused by wildfires and wildfire smoke on the reliability and longevity of PV modules in the field should also be explored.

## STAR★Methods

### Key resources table

**Table:** 

REAGENT or RESOURCE	SOURCE	IDENTIFIER
**Deposited data**		

Mean hourly PM2.5 concentration data across NSW	NSW Dept of Planning and Environment	https://www.dpie.nsw.gov.au/air-quality/air-quality-data-services/data-download-facility
Cumulative half hourly gross PV energy generation data	This paper	N/A

**Software and algorithms**		

Code for simulating the data analysis model	Zenodo repository	https://doi.org/10.5281/zenodo.8373326
Python version 3.8	Python Software Foundation	https://www.python.org
Jupyter Notebook	Project Jupyter	https://jupyter.org/

### Resource availability

#### Lead contact

Further information and requests should be directed to and will be fulfilled by the lead contact, Ethan Ford (ford_ethan@outlook.com).

#### Materials availability

This study did not generate new unique reagents.

#### Data and code availability

• This work analyses two datasets. The first is existing, publicly available data from the NSW Department of Planning and Environment data download facility. A link to this publicly available dataset is listed in the key resources table. The second is a private dataset procured by UNSW from Solar Analytics.
• All original code has been deposited at Zenodo and is publicly available as of the date of publication. DOIs are listed in the key resources table.
• Any additional information required to reanalyse the data reported in this work is available from the lead contact upon request.

### Method details

#### Data filtering

The datasets consisted of 30-minute cumulative energy data from 710 residential PV systems and 60-minute average PM_2.5_ concentrations from 50 meteorological sites across NSW from 6 Nov 2019 to 15 Jan 2020. A data filtering process was created in Python 3.8 to detect inverter clipping and non-generation (subpar performance or sensor error) in the PV generation data and exclude PV sites with such issues from further analysis.^31^

The method was tailored to our dataset but can be generalised to analyse similar PV datasets. Examples for used and discarded datasets are shown in below figure. PV generation data is given in units of Watt-hours (Wh) per 30-minute interval. Hence, doubling the value gives the mean power output of the PV system over that 30-minute period in W.

**Figure:**
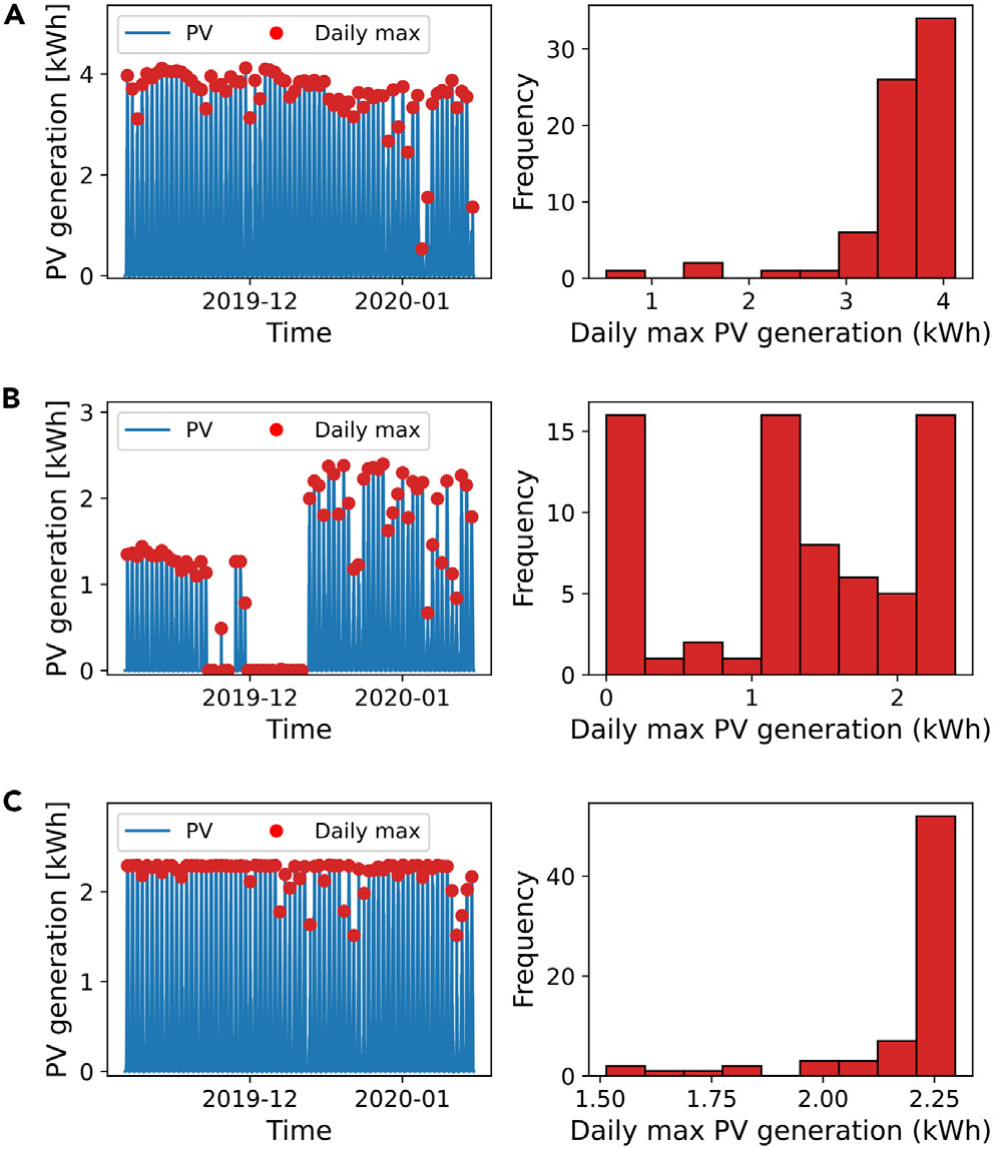
Comparison of data from typical PV sites This figure shows three examples of different performance characteristics in the PV dataset over the wildfire period from 6 Nov 2019–15 Jan 2020. Here *n* represents the number of PV sites falling into each category. Red dots over the raw data in the left column of plots indicate the maximum output for each day. The right column shows histograms of these daily maximum output and are used to assess whether a PV system is operating normally. (A) PV site operating as expected with raw data (left) and the frequency histogram of daily maximum output (right). (B) PV site with poor performance in early Nov and non-generation, possibly due to a sensor error, from late Nov to early Dec (left), resulting in a trimodal distribution (right). (C) PV site with inverter clipping, seen by the daily maximum output limited to approximately 2.3 kWh (left). This is confirmed by the histogram plot (right) with almost all daily maxima in the rightmost bin.

The plot (A) in the above figure represents a well-performing system in Australia. Typically, the Australian climate results in high PV generation with a small day-to-day variation resulting from changes in irradiance (e.g., clouds, wildfire smoke) and temperature, which is apparent in the histogram as well.

A first criterion for discarding data was to check for completion. If the number of data points for the PV site was not the expected number for the wildfire period considering 30-minute resolution, then the PV site had incomplete data and it was discarded. This happened for 5% of all sites. In addition to missing data, there were sensor errors resulting in a zero reading or a sensor error note when significant PV generation was expected. We classified a non-generation as any dataset in which:

1) The leftmost histogram bin of maximum PV generation contained more than 5 measurements—see (B) in the above figure.
2) The maximum PV generation was less than 100 Wh (equivalent to 200 W of power output).
3) The minimum daily maximum of PV generation was less than 5 Wh.

Such errors were present in 4% of all sites.

Inverter clipping is the artificial reduction of peak power output. An example of a clipped system is shown in plot (C) of the above figure. The high number of instances in the highest performance bin are indicative of a limitation in inverter output. Such systems are unsuitable for our analysis, as output power does not correlate with insolation. We classified clipping events as any dataset in which:

1) There were at least 5 consecutive PV data points (2 hour period) that are within 1% of the maximum PV recording of each other; and.
2) The 5 consecutive data points were all greater than 98% of the PV maximum for that site; and.
3) Both (1) and (2) occurred simultaneously at least 5 times during the period of the wildfires.

Clipping affected 16% of all sites, showing significant curtailment of PV output during the Australian summer.

The number of excluded sites for the different mentioned reasons are summarised in below table. More than ¾ (537) of the investigated systems were not affected by inverter clipping or missing data and were suitable for use in this study. Due to the high number of PV systems with suitable data, we could additionally filter out systems that were further away than 5 km from a PM_2.5_ monitoring site. We also excluded PM_2.5_ monitoring sites that were missing more than 5% of the air quality data for the period of interest. In total, we identified 160 sites that were included in this study, ranging in measured size from 0.5–5.4 kW with a mean of 2.0 kW and total capacity of 312 kW. System size was estimated as the maximum 80-percentile generation of each PV system under negligible air pollution — i.e. the peak of the green clear sky curve in Figure 3.

**Table:** Categorization results from data filtering

Criteria	Number of PV sites	Percentage of total PV sites (%)
Total PV sites	710	100
Missing data	36	5.07
Inverter clipping	115	16.2
Non-generation	28	3.94
Clean data	537	75.6
Correlated with PM_2.5_	160	22.5

#### Geospatial analysis

Sites that passed the above filtering process are plotted in below figures. Geographical coordinates were available for the PM_2.5_ sites, but PV site locations were only accurate to the mean coordinates of their respective postcodes as the street address was not known to us due to privacy reasons.^32^ Postcodes in Australia are usually irregularly shaped regions containing cities, suburbs, or towns. The area of postcodes is smaller in highly populated regions. This means the location of the PV sites is relatively precise for metropolitan areas and less accurate for rural areas.

**Figure:**
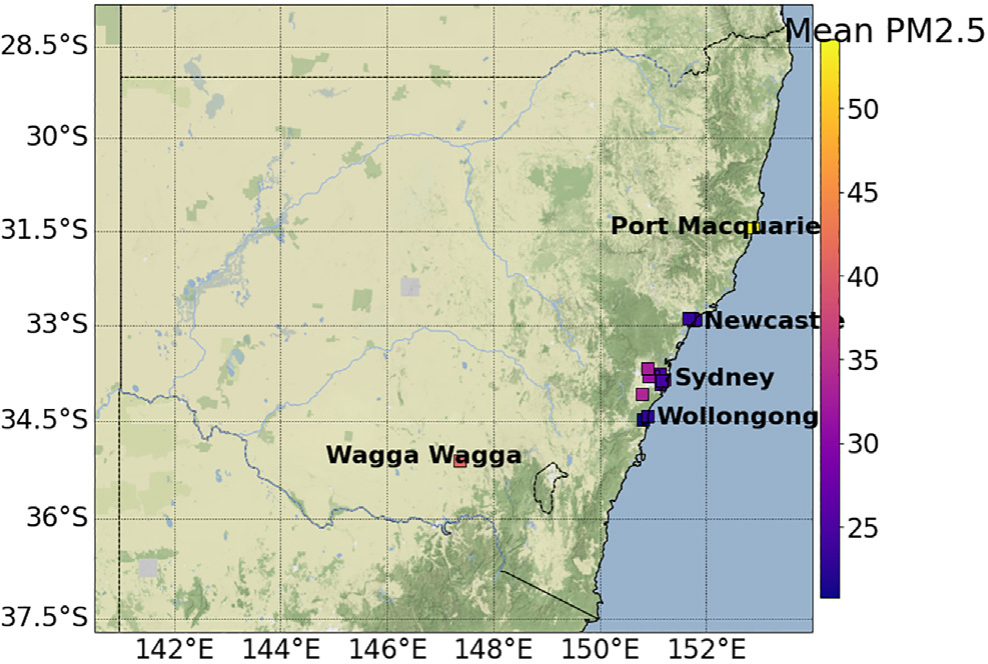
Locations of PM_2.5_ monitoring sites in NSW This figure uses colored squares to map the locations of all PM_2.5_ monitoring sites used in the analysis. The color indicates the mean daytime PM_2.5_ concentrations for the wildfire period in μg/m^3^. This gives an indication of the total amount of smoke in specific regions across NSW during the wildfires, but it does not show the variability in smoke concentration throughout that period. Port Macquarie has the highest mean daytime PM_2.5_ concentration, to which the smoke plumes on 8 Nov 2019 contributed (similar to Figure 1).

**Figure:**
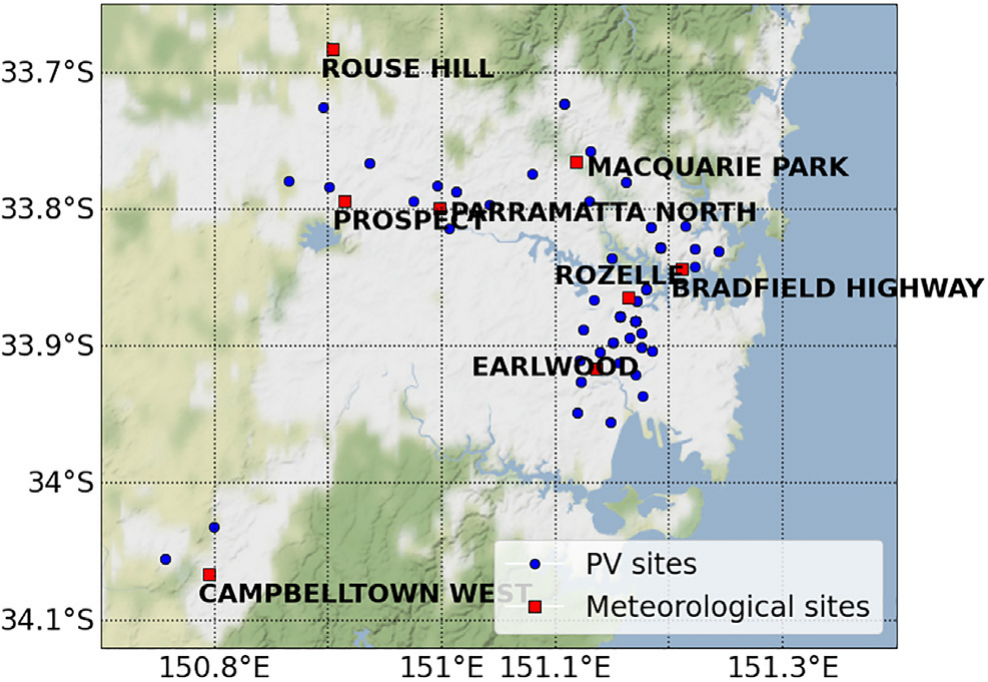
Distribution of PV and PM_2.5_ sites in Sydney Blue dots indicate postcodes that contain at least one PV site with clean data (see table in data filtering section) that are within 5 km of a meteorological site (red squares). The Sydney area is the most densely populated region in this analysis.

The distance and direction between PV sites and PM_2.5_ monitors is expected to substantially influence the correlation between the two sites—this is discussed in the Correlation between distance and reduction rate section.

#### 80-Percentile clear sky filter

PV generation data was sorted into a two-dimensional array of bins according to the hour of the day and PM_2.5_ concentration. As the temporal resolution of the PV data was twice that of the PM_2.5_ data, the PV data was binned in pairs (e.g. 30-minute and 60-minute). The 80-percentile of each bin was used to represent clear sky (sunny) conditions for each PM_2.5_ range, as shown in Figure 3. The value for the percentile filter was empirically derived and used by Peters et al.^23^^,^^30^ The 80-percentile value removes instances with cloud and rain where the PV generation is low, and instances due to cloud enhancement effects on partially cloudy days where PV generation exceeds that on a clear sky day. Due to changes in the wind direction and intensity of the wildfires, we had intermittent hazy and clear days for most sites. In addition, this approach intrinsically corrects for soiling of the PV module as soiling can be assumed to affect all PM_2.5_ bins equally.
