# Supplementary material for: Quantifying the impact of wildfire smoke on solar photovoltaic generation in Australia
Source: iScience. 2023 Nov 30;27(2):108611. doi: 10.1016/j.isci.2023.108611 (PMC10845029; doi:10.1016/j.isci.2023.108611)
Supplement: Document S1. Figure S1 [file mmc1.pdf]

iScience, Volume *27*

Supplemental information

Quantifying the impact of wildfire smoke on solar photovoltaic generation in Australia

Ethan Ford, Ian Marius Peters, and Bram Hoex

Supplementary Information

This document contains supplementary information for the results in Fig. 7 of the article on how air pollution reduces PV generation for a single PV site over the course of a typical day. We investigated whether the result we see for one PV site—that the reduction in PV generation is greater in the morning—was applicable generally. The below Fig. S1 was derived directly from Fig. 8 in the article which illustrates how air pollution reduces PV generation for 160 PV sites in NSW. We partitioned the data by hour to find the reduction rate for all 160 PV systems for each hour of daylight and the results are plotted in orange. To visualise the effect of the air mass-correction on these results, we repeated this process for raw PM2.5 concentrations uncorrected for air mass (AM), which are plotted in blue. We observe the impact is roughly symmetrical either side of solar noon—the outlier being 9 am—with the greatest reductions in the morning around 10 am and in the evening from 4 pm onwards.

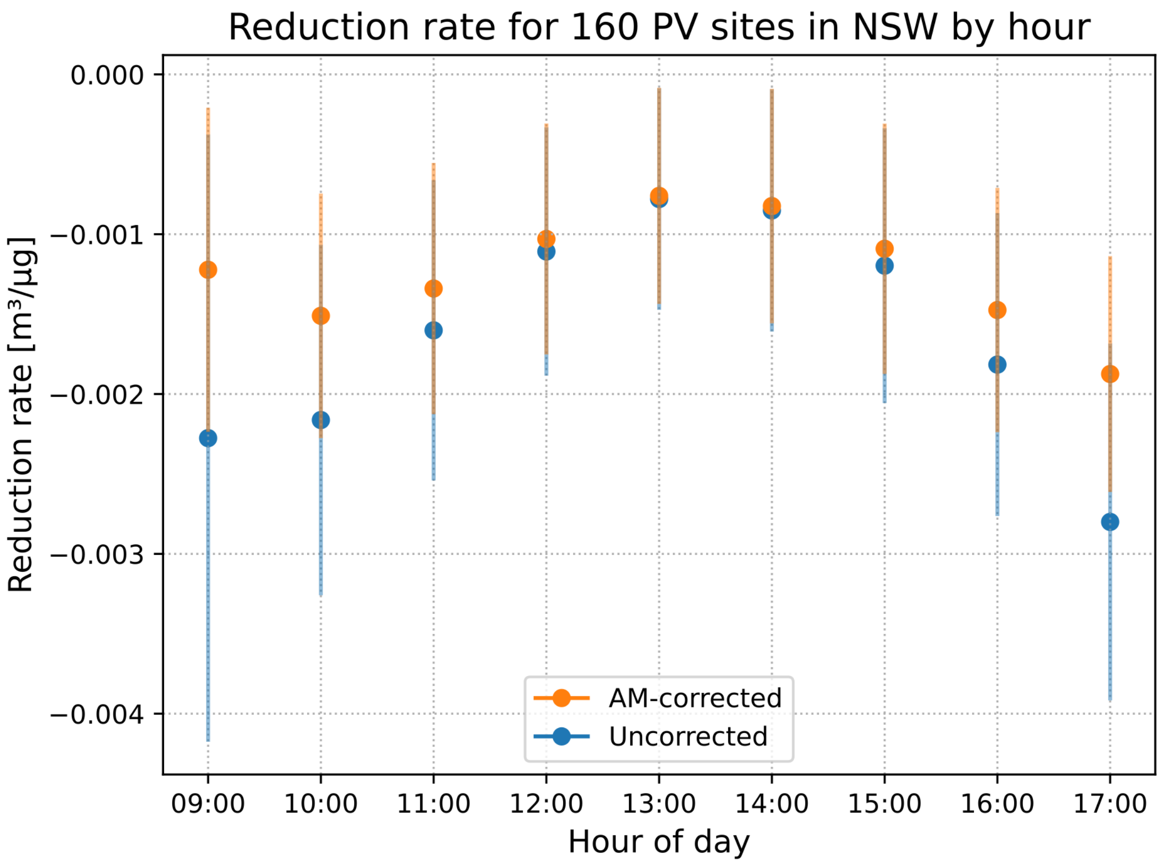

Fig. S1 How air pollution reduces PV generation for 160 PV sites in NSW over the course of a typical day. Related to Fig. 7. PV reduction rate for AM-corrected PM2.5 concentration (orange) and uncorrected PM2.5 concentration (blue) plotted against the hour of the day. The difference in profile and error bar size suggests that the AM-correction improves the analysis as it reduces the spread in the result for each hour. However, the hour-to-hour variation indicates that the AM-correction is not able to fully account for the trend we see.
